# Aortic Function in a Longitudinal 4D Flow MRI Study in Marfan Syndrome Patients Receiving Resveratrol

**DOI:** 10.1002/jmri.70021

**Published:** 2025-06-30

**Authors:** Daan Bosshardt, Mitzi M. van Andel, E. M. Schrauben, Roland R. J. van Kimmenade, Arthur J. Scholte, Moniek G. J. P. Cox, Daniëlle Robbers‐Visser, Aeilko H. Zwinderman, Barbara J. M. Mulder, Aart J. Nederveen, Vivian de Waard, Maarten Groenink, Pim van Ooij

**Affiliations:** ^1^ Department of Radiology & Nuclear Medicine Amsterdam UMC Amsterdam the Netherlands; ^2^ Department of Cardiology Amsterdam UMC Amsterdam the Netherlands; ^3^ Amsterdam Cardiovascular Sciences Atherosclerosis & Ischemic Syndromes Amsterdam the Netherlands; ^4^ Department of Medical Biochemistry Amsterdam UMC Amsterdam the Netherlands; ^5^ Department of Cardiology St. Radboud University Hospital Nijmegen the Netherlands; ^6^ Department of Cardiology Leiden University Medical Center Leiden the Netherlands; ^7^ Department of Cardiology University Medical Center Groningen Groningen the Netherlands; ^8^ Department of Clinical Epidemiology, Biostatistics and Bioinformatics Amsterdam UMC Amsterdam the Netherlands

**Keywords:** 4D flow MRI, arterial stiffness, Marfan, resveratrol, wall shear stress

## Abstract

**Background:**

New treatment strategies are required to reduce aortic events in Marfan syndrome (MFS). Resveratrol is a dietary supplement that intervenes in aortic wall cellular metabolism and may benefit MFS patients.

**Purpose:**

To evaluate whether treatment with Resveratrol affects aorta hemodynamics derived from 4D flow MRI in MFS.

**Study Type:**

Prospective single‐arm open‐label multicenter trial.

**Population:**

46 MFS patients (mean age 36 ± 9 years, 23 female), 20 with and 26 without a history of aortic root surgery (RR and native MFS), and 25 age‐ and sex‐matched healthy controls.

**Field Strength/Sequence:**

3T, temporally resolved 3D phase contrast (4D flow MRI) and 3D mDixon sequences.

**Assessment:**

MFS patients underwent 4D flow MRI before (baseline) and after 1 year of 500 mg daily Resveratrol treatment (follow‐up). Velocity magnitude and wall shear stress (WSS) in six regions in the thoracic aorta were evaluated. Incidence maps showing abnormal hemodynamics compared to healthy controls were generated. Pulse wave velocity (PWV) was assessed in 45 subjects. The relationships between MRI parameters and annual aortic growth from the 3D mDixon scans and age were investigated.

**Statistical Tests:**

Student's paired and unpaired t‐tests, Fisher's exact tests, McNemar's exact test, and Pearson correlation coefficients (r). Statistical significance was defined as *p < 0.05*.

**Results:**

No significant changes in any of the hemodynamic parameters were observed: inner descending aorta WSS (Pa), native: from 1.08 ± 0.21 to 1.08 ± 0.17, *p = 0.818*, RR: from 1.02 ± 0.29 to 0.98 ± 0.21, *p* = 0.270; velocity (m/s), native: from 0.64 ± 0.10 to 0.65 ± 0.10, *p* = 0.359, RR: 0.68 ± 0.15, to 0.67 ± 0.12, *p* = 0.629; abnormally directed WSS incidence: native: from 17 (65%) to 14 (54%), *p* = 0.453, RR: 13 (65%) to 13 (65%), *p* = 1.000; PWV (m/s) native: from 7.7 ± 1.9 to 7.9 ± 1.8, *p* = 0.582, RR: 9.2 ± 1.6 to 8.2 ± 3.2, *p* = 0.184. PWV correlated with age in RR MFS patients (*r* = 0.59, *n* = 19).

**Data Conclusion:**

No significant changes in aortic hemodynamics derived from 4D flow MRI were observed after 1 year of Resveratrol treatment.

**Evidence Level:**

1.

**Technical Efficacy:**

Stage 4.

**Summary**

- Plain language summary
  ○ Resveratrol, a dietary supplement, has recently gained interest for Marfan syndrome as it may influence aortic wall metabolism and may slow down the aortic dilation rate.
  ○ This study aimed to evaluate whether Resveratrol affects aortic blood flow parameters, measured using 3D phase contrast MRI, as an indirect marker of changes in arterial cellular processes.
  ○ Forty‐six Marfan syndrome patients were treated with 500 mg of Resveratrol daily for 1 year.
  ○ No significant changes in hemodynamic parameters or incidence of abnormal flow were found after treatment, suggesting that Resveratrol does not substantially alter aortic hemodynamics.

## 1 Introduction

An acute aortic syndrome is a detrimental manifestation of Marfan syndrome (MFS) [1]. Aortic dilatation often, but not always, precedes aortic events, and is thus of limited value in guiding appropriate timing of elective aortic surgery [2, 3, 4]. Current treatment strategies, beta blockers, and angiotensin‐II receptor blockers have been shown to induce a modest decrease in aortic dilatation rate [5, 6]. New treatment strategies to not only decrease dilatation rate but also decrease the incidence of aortic events by modifying aortic wall properties are warranted. A single‐arm open‐label multicenter trial investigating the effects of Resveratrol on aortic growth rate in a Marfan syndrome cohort (the RESVcue trial [7]) has recently been conducted. In this study, there was a trend toward a decrease in aortic root growth rate after 1 year of 500 mg daily Resveratrol administration, showing promise for conducting a subsequent randomized controlled trial. Resveratrol has been shown to modify endothelial dysfunction, extracellular matrix degradation, and smooth muscle cell death in MFS mice and other rodent models [8, 9, 10]. These changes in the arterial wall properties might improve aortic hemodynamics.

Four‐dimensional (4D) flow MRI allows for the non‐invasive evaluation of aortic hemodynamic parameters by assessing aortic flow through the cardiac cycle in a 3D geometry [11]. The technique also allows the evaluation of wall shear stress (WSS) and flow velocity and direction, together with pulse wave velocity (PWV), an indirect measurement of arterial stiffness [12].

4D flow MRI parameters are potential biomarkers for aortic disease, since abnormal WSS and altered aortic flow patterns have been observed in MFS [13, 14]. However, there have been few studies investigating changes in 4D flow MRI parameters over time in MFS [15, 16], and studies on changes after administration of Resveratrol are currently lacking.

Thus, the aim of this study, which was a sub‐study of the RESVcue trial, was to investigate whether 4D flow MRI parameters change after 1 year of Resveratrol treatment.

## 2 Materials and Methods

### 2.1 Study Design and Participants

The local ethics boards approved this study, and written informed consent was obtained from all participants. Patients were prospectively included to undergo an MRI examination before and after 500 mg daily Resveratrol treatment (EHF Production, Rotterdam, The Netherlands), which was started the same day, until 1 year after this starting point. Patients were enrolled from December 2018 to November 2020. Patients were identified by four university hospitals with a specialized multidisciplinary Marfan screening clinic and by using the CONgenital CORvita (CONCOR) Dutch national registry for adult congenital heart disease [17]. Eligible patients were adults (≥ 18 years) who were diagnosed with MFS according to the revised Ghent criteria, with a known pathogenic FBN1 gene variant and at least one MRI scan of their aortic trajectory > 1 year prior to the current study [18]. Patients were ineligible if they (i) had an aortic root diameter > 50 mm, (ii) had a history of aortic dissection, (iii) had more than one vascular prosthesis, (iv) had aortic surgery in the last 6 months before inclusion or (v) were considered likely to undergo aortic surgery within 6 months of inclusion, as determined by the treating physician. Moreover, patients were ineligible if they (vi) were known to have a neurodevelopmental disorder or (vii) had the intention to become pregnant in the following year. Results were reported separately for patients with and without a history of aortic root surgery (RR and native MFS, respectively).

### 2.2 MR Imaging

All patients underwent 3 Tesla MR (Ingenia, Philips, Best, The Netherlands) examinations using a 16‐channel anterior and 12‐channel posterior coil before (baseline) and after 1 year of Resveratrol (follow‐up). The aorta was imaged using a non‐contrast‐enhanced ECG‐gated and respiratory navigator‐gated 3D mDixon sequence targeted at mid‐diastole and end‐expiration with spatial resolution acquired: 1.25 × 1.25 × 1.25 mm, reconstructed: 0.73 × 0.73 × 0.625 mm, repetition time, echo time 1 & 2, flip angle(TR/TE1/TE2/FA): 5.2/1.93/3.4 ms/15°, field of view (FOV): 315 × 274.2 × 60 mm, 96 slices stack, slice orientation: sagittal.

Aortic diameters were assessed on five predefined cross‐sections of the mDixon water images: aortic root at the level of the sinus Valsalva; the ascending aorta (AAo) at the level of the pulmonary artery bifurcation or superior to the aorta graft in patients with a history of aortic root replacement; the aortic arch in between the left carotid and left subclavian artery; the proximal descending aorta (DAo) at the level of the pulmonary artery bifurcation and the distal DAo at the level of the diaphragm. The largest diameter measured at each level was used for analysis. To acquire the diameter measurements, all initial measurements were performed by MMvA (4 years of experience). Subsequently, the positioning and acquisition of the diameter measurements were reviewed by DB (3 years of experience) to ensure consistency between MRI scans. In case of disagreement regarding position or diameter, MG (30 years of experience) decided on the final measurement. The repeatability of the current diameter measurements was previously investigated and was found to be very good [7].

An in‐house developed non‐contrast enhanced prospectively ECG‐ and end‐expiratory respiratory navigator gated 3D phase contrast MRI (4D flow MRI) acquisition was performed with parameters: acquired and reconstructed spatial resolution: 2.5 × 2.5 × 2.5 mm^3^; (TR/TE/FA) 3.9 ms/2.1 ms/8°; velocity encoding: 150–250 cm/s, FOV: 315 × 276 × 60 mm, 24 slices stack, slice orientation: sagittal. A variable density pseudo‐spiral Cartesian pattern was sampled with PROspective Undersampling in multiple Dimensions (PROUD) as previously described, with a target acceleration factor *R* = 8 [19]. The median scan time was 3.31 min (range: 3.24–3.91), depending on the extent of arrhythmia rejection. Images were reconstructed into 30 timeframes with a median temporal resolution of 32 ms (range: 24–45), depending on heart rate.

### 2.3 Image Processing

**FIGURE 1:**
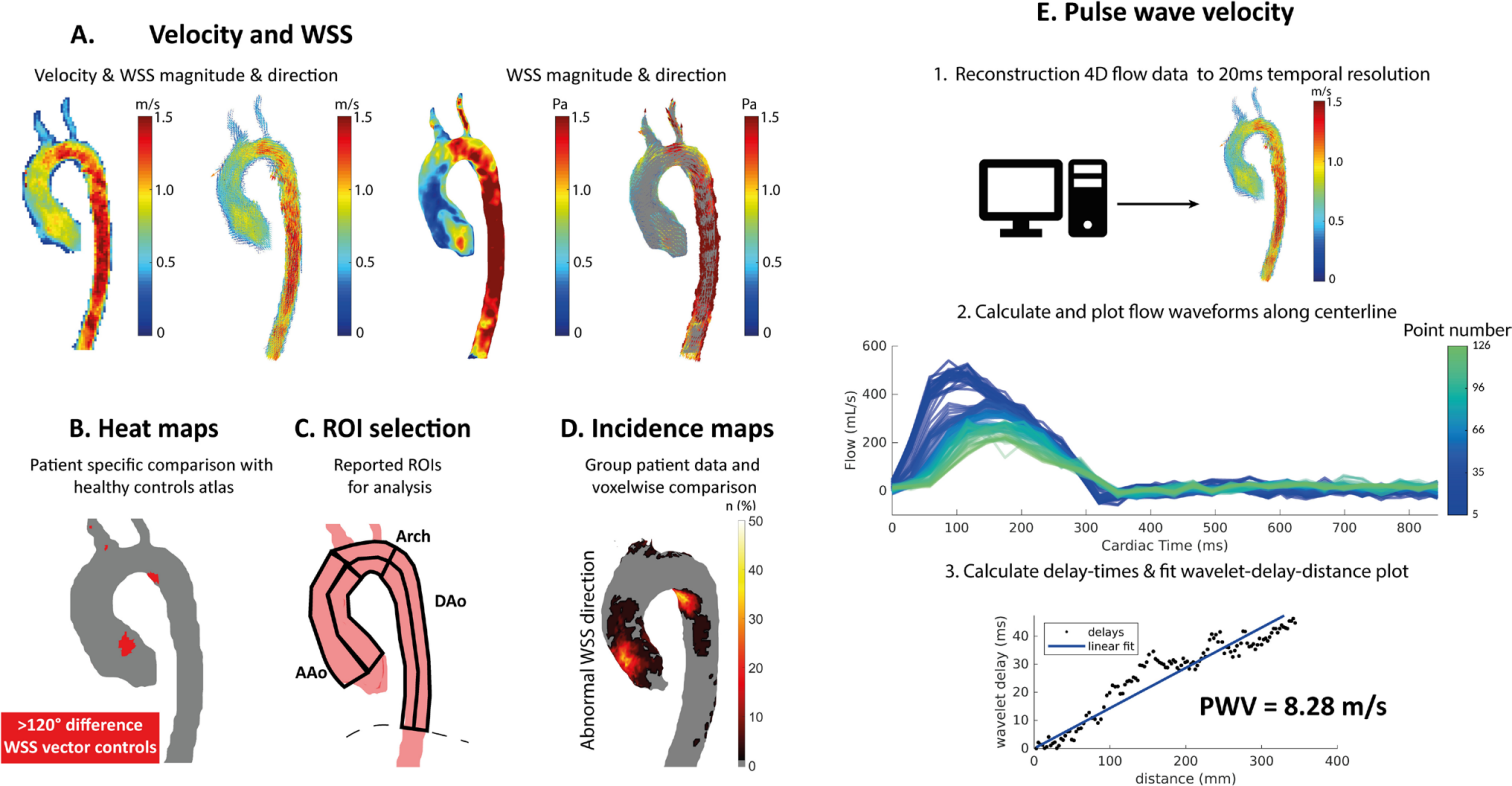
Overview of the acquired 4D flow MRI parameters: (A) Velocity and wall shear stress (WSS) magnitude for each patient and quantification of mean values in the inner and outer ascending aorta, inner and outer arch, and inner and outer descending aorta. Additionally, velocity and WSS direction quantification per patient. (B) Comparison of magnitude and direction of hemodynamics with an atlas of age‐ and sex‐matched healthy volunteers, and creation of heat maps showing voxels with abnormal hemodynamics. (C) Selection of six regions of interest in the thoracic aorta for quantitative hemodynamic analysis. (D) Projection of all abnormal hemodynamic heat maps onto a shared geometry, creating incidence maps that identify regions with a high incidence of abnormal hemodynamics within each patient group. (E) Reconstruction of 4D flow MRI data to 20 ms temporal resolution, followed by centerline calculation and extraction of flow waveforms. Lastly, wavelet delay time calculation using wavelet cross‐spectrum analysis and pulse wave velocity (PWV) calculation by fitting the delay‐distance plot.

All 4D flow MRI scans were reconstructed using an in‐house developed compressed sensing pipeline using the Berkeley Advanced Reconstruction Toolbox (BART, Berkeley, California), Matlab (The Mathworks Inc., Natick, MA, USA), and ReconFrame (Gyrotools, Zurich, Switzerland) [19, 20]. An overview of all acquired 4D flow MRI parameters is provided in Figure 1. All velocity data were automatically corrected for background phase offsets (Reconframe). The aorta was manually segmented on time‐averaged phase contrast MR angiogram images (phase contrast magnitude images multiplied by absolute velocity) by threshold, watershed, and manual voxel inclusion/exclusion in Mimics (Materialize, Leuven, Belgium). Aorta segmentations were performed by MMvA (4 years of experience) and reviewed by DB (3 years of experience). The segmentations were used to mask the velocities and calculate WSS as previously described [21]. In short, the WSS vectors were estimated at the aortic wall based on the 3D spatial velocity gradient perpendicular to the vessel wall, using three points along the inward normal of the vessel wall, spanning 50% of the lumen diameter. 3D WSS magnitude was calculated at peak systole, defined as the timeframe in which the spatially averaged velocity magnitude within the segmented aortic volume was highest. Mean peak systolic WSS and velocity per region were reported and used for all analyses (Figure 1A).

Six regions in the thoracic aorta were defined: the inner and the outer AAo, from aortic root or the equivalent level in the graft of RR MFS patients, to brachiocephalic trunk; the inner and outer aortic arch, from brachiocephalic trunk to the left subclavian artery; and the inner and outer DAo, extending from the left subclavian artery to the level of the diaphragm (Figure 1C).

Regions were drawn by MMvA (4 years of experience). Additionally, DB (3 years of experience) independently drew the regions of interest and calculated WSS and velocity to assess inter‐observer variability.

To ensure consistent regions of interest between consecutive scans, rigid registration of the follow‐up 4D flow MRI segmentation to the baseline segmentation was performed for each subject using FLIRT (FMRIB's Linear Image Registration Tool [22]).

4D flow MRI data, which had previously been acquired for a prospective study of twenty‐five healthy, age‐ and sex‐matched subjects, were used to create an atlas of normal values for flow velocity and WSS [23]. Briefly, these atlases represent the 95% confidence interval (CI) (mean ± 1.96 × SD) of the velocity and WSS values at each voxel, generated by interpolating the absolute velocity fields of healthy volunteers onto a shared aortic geometry, and the individual WSS magnitudes onto the wall points of this geometry. Next, the 3D segmentation of each MFS aorta was co‐registered to the shared aortic geometry, and the velocity and WSS atlases were interpolated onto the aortic segmentation of the individual MFS patient. Heatmaps identifying regions with elevated velocity and WSS could subsequently be created where values were higher than the 95% CI of the atlases (Figure 1B) [24]. Similar to this methodology to assess elevated velocity or WSS magnitude, normal‐averaged velocity and WSS atlases were created to create heatmaps identifying abnormal vectors [25]. Abnormally directed velocity and WSS were defined as vector angle differences higher than 120° compared to the healthy subject atlases [25]. A patient was considered to have abnormal hemodynamics when at least one voxel with abnormal hemodynamics was present and was evaluated for each region of interest separately. Maps of abnormal hemodynamics of each MFS patient were subsequently projected on cohort‐specific “shared” patient geometries to create 3D incidence maps visualizing the prevalence of abnormal hemodynamics per voxel for each patient group (Figure 1D) [23, 26].

To ensure sufficient temporal resolution for PWV calculation, all MFS 4D flow MRI scans were reconstructed a second time to a fixed 20 ms temporal resolution, and using an iterative background phase correction method [27]. PWV measurements were performed using a previously described open‐source Matlab‐based app (Figure 1E) (R2021b, Mathworks, MA, USA) [28]. Briefly, the tool performs data and segmentation loading and aortic centerline extraction [29]. Combined with the aortic segmentations, orthogonal cross‐sections extracted from the centerline were used to measure flow along the aorta automatically. PWV values were subsequently calculated after determining the delay times of the flow waveforms using a wavelet cross‐spectrum analysis and performing linear regression through the delay‐distance plot [30].

### 2.4 Other Measurements

Brachial blood pressure was measured before every MRI examination. Mean arterial pressure (MAP) was defined as: (2 * diastolic blood pressure + systolic blood pressure)/3. Reports from the most recent clinically acquired transthoracic echocardiograms were assessed to determine the presence of aortic regurgitation.

### 2.5 Statistical Analysis

Statistical analyses were performed using R version 4.3.2 (R Foundation for Statistical Computing, Vienna, Austria). Categorical data were reported as numbers and percentages. Continuous data were reported as mean ± standard deviation (SD) or median and interquartile range (IQR), depending on data distribution as assessed by visual inspection of histograms. Comparison of continuous variables was performed with a Student's paired t‐test for consecutive continuous measurements and an independent‐samples t‐test for group comparisons, both accompanied by 95% CI. Fisher's exact tests were used to test for group differences in nominal variables, and the incidence of abnormal hemodynamics was compared between baseline and follow‐up using McNemar's exact test. In an exploratory analysis, Pearson correlation coefficients (r) were used to assess the relationships between baseline or change in 4D flow MRI parameters and locoregional aortic growth (diameter increase per year) and age. Inter‐observer variability of the WSS and velocity parameters was assessed using the intraclass correlation coefficient (ICC) with 95% confidence intervals, based on a two‐way random effects model for absolute agreement, and absolute differences were reported. A *p*‐value < 0.05 was considered significant.

## 3 Results

Of the 57 patients enrolled in the RESVcue trial, 4D flow MRI data were available in 46 patients. The median follow‐up duration was 371 days (IQR: 364–453). In 11 (19%) patients, 4D flow MRI could not be performed or completed due to logistical or technical constraints. The baseline characteristics of the remaining 26 native MFS and 20 RR MFS patients are presented in Table 1. RR MFS patients were significantly more likely to be male (75% vs. 31%) and had a significantly higher body surface area (2.2 ± 0.2 m^2^ vs. 2.0 ± 0.2 m^2^). MAP and heart rate did not differ between baseline and follow‐up for native (MAP (mmHg): from 82 ± 9 to 82 ± 8, *p = 0.835*; heart rate (bpm): from 61 ± 9 to 62 ± 11 *p* = 0.860) and RR MFS patients (MAP (mmHg): from 84 ± 10 to 83 ± 8, *p = 0.823*, heart rate (bpm): from 60 ± 10 to 62 ± 8, *p = 0.576*).

**TABLE 1:** Demographics and clinical characteristics of the patients with Marfan syndrome without and with a history of aortic root surgery.

	Native MFS, *n* = 26	RR MFS, *n* = 20
*General features*		
Age at inclusion (years)	36 ± 9	37 ± 9
Sex (female)	18 (69%)	5 (25%)*
Body surface area (m^2^)	2.0 ± 0.2	2.2 ± 0.2*
FBN1 mutation		
Dominant‐negative	10 (39%)	10 (50%)
Haploinsufficient	12 (46%)	9 (45%)
Unknown effect	4 (15%)	1 (5%)
Cardiac medication		
β‐blocker	16 (61%)	13 (65%)
Losartan	18 (69%)	12 (60%)
β‐blocker and Losartan	13 (50%)	8 (40%)
Systolic BP (mmHg)	114 ± 13	117 ± 14
Diastolic BP (mmHg)	66 ± 9	67 ± 11
MAP (mmHg)	82 ± 9	84 ± 10
≥ grade I aortic insufficiency	0 (0%)	6 (30%)

*Note*: *p‐value < 0.05 comparing frequencies or mean values between Marfan syndrome patients with native and operated aortic root.

Abbreviations: BP, blood pressure; MAP, mean arterial pressure; Native MFS, Marfan syndrome patient with a native aortic root; RR MFS, Marfan syndrome patient with a history of aortic root surgery.

An overview of aortic diameters and aortic growth per aortic region, as assessed on the mDixon scan, is presented in Table 2. Aortic ascending, arch, proximal descending, and distal descending diameters were significantly higher in the RR MFS group compared to the native MFS at both baseline and follow‐up. No significant differences were observed in aortic growth rates between native and RR MFS patients (Table 2). Aortic diameters did not change significantly between baseline and follow‐up in either patient group (Table S1).

**TABLE 2:** Aortic diameters and growth rates at baseline and follow‐up.

Aortic level	Native MFS (*n* = 26)	RR MFS (*n* = 20)	Difference, Mean [95% CI]	*p*, native vs. RR MFS
	Mean ± SD	Mean ± SD		
*Baseline (mm)*				
Root	41.4 ± 4.6	—	*—*	*—*
Ascending	29.2 ± 2.6	31.8 ± 2.4	2.6 [1.0, 4.2]	***0.002***
Arch	24.9 ± 2.1	28.6 ± 2.4	4.1 [2.7, 5.6]	***< 0.001***
Proximal descending	24.6 ± 2.6	28.0 ± 4.3	3.1 [1.1, 5.1]	***0.003***
Distal descending	20.6 ± 2.1	23.6 ± 1.7	2.8 [1.7, 4.0]	***< 0.001***
*Follow‐up (mm)*				
Root	41.1 ± 4.7	—		
Ascending	29.2 ± 2.5	31.8 ± 2.4	2.6 [1.1, 4.1]	***0.001***
Arch	25.2 ± 2.4	28.6 ± 2.4	3.4 [2, 4.9]	***< 0.001***
Proximal descending	24.5 ± 2.6	28.0 ± 4.3	3.6 [1.5, 5.7]	***0.001***
Distal descending	20.7 ± 2.0	23.6 ± 1.7	2.9 [1.8, 4.0]	***< 0.001***
*Annual growth rate (mm/year)*				
Root	−0.2 ± 1.2	—	*—*	*—*
Ascending	0.0 ± 1.3	−0.1 ± 1.3	−0.1 [−0.9, 0.7]	*0.811*
Arch	0.2 ± 1.1	−0.1 ± 1.2	−0.4 [−1.1, 0.3]	*0.279*
Proximal descending	−0.1 ± 1.2	0.3 ± 2.0	0.5 [−0.5, 1.4]	*0.335*
Distal descending	0.1 ± 1.2	0.2 ± 1.1	0.1 [−0.6, 0.7]	*0.864*

*Note: p*‐values < 0.05 were considered significant (italic and bold italic).

Abbreviations: Native MFS, Marfan syndrome patient with a native aortic root; RR MFS, Marfan syndrome patient with a history of aortic root surgery.

An overview of 4D flow MRI parameters is provided in Table 3 and shows no significant differences between baseline and follow‐up velocities. Velocities (m/s) in the outer AAo and outer arch were significantly higher for RR MFS patients compared to native MFS at both baseline (outer AAo: 0.80 ± 0.15 vs. 0.50 ± 0.09; outer arch: 0.65 ± 0.14 vs. 0.53 ± 0.09) and follow‐up (outer AAo: 0.79 ± 0.14 vs. 0.49 ± 0.10; outer arch: 0.65 ± 0.14 vs. 0.53 ± 0.09). No significant differences in velocities between native and RR MFS patients were observed in the other aortic regions at either baseline or follow‐up (Table S2).

**TABLE 3:** 4D flow MRI parameters at baseline and follow‐up.

	Native MFS (*n* = 26)				RR MFS (*n* = 20)			
	Baseline	Follow‐up	Change	*p*	Baseline	Follow‐up	Change	*p*
	Mean ± SD	Mean ± SD	Mean [95% CI]		Mean ± SD	Mean ± SD	Mean [95% CI]	
*Velocity (m/s)*								
Inner AAo	0.61 ± 0.11	0.61 ± 0.11	0.00 [−0.03, 0.03]	*0.896*	0.68 ± 0.14	0.67 ± 0.16	−0.01 [−0.07, 0.04]	*0.686*
Outer AAo	0.50 ± 0.09	0.49 ± 0.10	0.00 [−0.03, 0.02]	*0.712*	0.80 ± 0.15	0.79 ± 0.14	−0.02 [−0.05, 0.02]	*0.327*
Inner arch	0.61 ± 0.09	0.61 ± 0.11	0.00 [−0.03, 0.03]	*0.976*	0.62 ± 0.12	0.62 ± 0.10	0.00 [−0.04, 0.03]	*0.831*
Outer arch	0.53 ± 0.09	0.53 ± 0.09	0.00 [−0.03, 0.04]	*0.779*	0.65 ± 0.14	0.65 ± 0.14	−0.01 [−0.05, 0.03]	*0.739*
Inner DAo	0.64 ± 0.10	0.65 ± 0.10	0.01 [−0.01, 0.04]	*0.359*	0.68 ± 0.15	0.67 ± 0.12	−0.01 [−0.04, 0.03]	*0.629*
Outer DAo	0.68 ± 0.12	0.69 ± 0.12	0.00 [−0.03, 0.04]	*0.925*	0.68 ± 0.13	0.7 ± 0.14	0.02 [−0.01, 0.05]	*0.223*
*WSS (Pa)*								
Inner AAo	0.70 ± 0.14	0.69 ± 0.15	−0.01 [−0.06, 0.04]	*0.735*	0.74 ± 0.21	0.72 ± 0.23	−0.01 [−0.11, 0.08]	*0.807*
Outer AAo	0.59 ± 0.15	0.57 ± 0.15	−0.01 [−0.05, 0.02]	*0.345*	0.95 ± 0.23	0.91 ± 0.21	−0.04 [−0.09, 0.01]	*0.139*
Inner arch	0.82 ± 0.14	0.81 ± 0.15	−0.01 [−0.06, 0.04]	*0.776*	0.80 ± 0.20	0.78 ± 0.15	−0.02 [−0.08, 0.04]	*0.437*
Outer arch	0.71 ± 0.13	0.71 ± 0.15	0.00 [−0.05, 0.06]	*0.883*	0.80 ± 0.21	0.79 ± 0.17	−0.01 [−0.06, 0.05]	*0.745*
Inner DAo	1.08 ± 0.21	1.08 ± 0.17	0.01 [−0.05, 0.06]	*0.818*	1.02 ± 0.29	0.98 ± 0.21	−0.04 [−0.11, 0.03]	*0.270*
Outer DAo	1.09 ± 0.24	1.08 ± 0.21	0.00 [−0.07, 0.06]	*0.922*	0.97 ± 0.24	1.00 ± 0.26	0.03 [−0.03, 0.10]	*0.255*
Pulse wave velocity (m/s)	7.7 ± 1.9	7.9 ± 1.8	0.2 [−0.5, 0.9]	*0.582*	9.2 ± 1.6^a^	8.2 ± 3.2^a^	−1.0 [−2.6, 0.5]^a^	*0.184*

*Note: p*‐values < 0.05 were considered significant (italic and bold italic).

Abbreviations: AAo, ascending aorta; DAo, descending aorta; Native MFS, Marfan syndrome without a history of aortic root surgery; RR MFS, Marfan syndrome with a history of aortic root surgery; WSS, wall shear stress.

^a^ *n* = 19 for RR MFS patients due to missing data from one patient.

No significant differences between baseline and follow‐up WSS parameters were observed (Table 3). Mean WSS (Pa) was significantly higher for RR versus native MFS patients in the outer AAo at both baseline (0.95 ± 0.23 vs. 0.59 ± 0.15) and follow‐up (0.91 ± 0.21 vs. 0.57 ± 0.15). No significant differences in WSS between native and RR MFS patients were observed in the other aortic regions at either baseline or follow‐up after Resveratrol treatment (Table S3).

The ICC values for velocity and WSS measurements were both 0.972 (95% CI: 0.965–0.978), with absolute differences of 0.023 ± 0.024 m/s for velocity and 0.023 ± 0.024 Pa for WSS indicating excellent reliability between observers.

**FIGURE 2:**
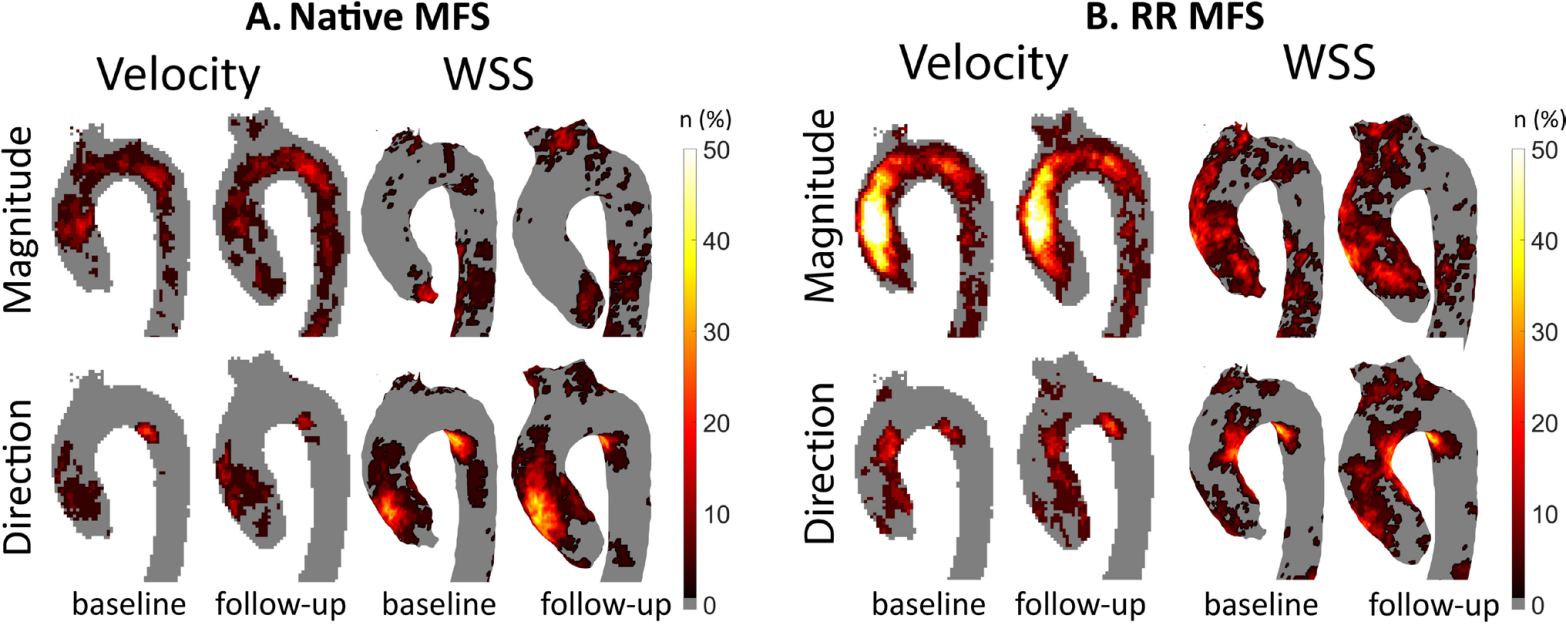
Incidence maps showing the proportion of patients (%) with abnormally elevated or directed velocity and wall shear stress (WSS) before and after Resveratrol administration compared to age & sex matched healthy aortas, on a per‐voxel basis in (A) Marfan syndrome (MFS) patients without a history of aortic root surgery (native) and (B) patients with a history of aortic root surgery (RR).

An overview of the comparison of the proportion of abnormal hemodynamics at baseline and follow‐up is presented in Table 4. None of the evaluated parameters showed a significant change in incidence. Incidence maps for abnormal aortic hemodynamics per patient group are shown in Figure 2. Comparing baseline to follow‐up incidence maps, abnormally elevated and directed velocity and WSS were present in both native and RR MFS patients. The localization of the elevated velocity and WSS was more pronounced in the AAo of RR MFS patients as compared to native MFS patients, with over 50% of the RR MFS patients exhibiting elevated flow in this region. Moreover, in the native MFS aortas, the direction of WSS in the AAo was altered, while this was not abnormal in the RR MFS aortas. Both native and RR MFS patients showed a region of abnormally directed velocity and WSS in the proximal inner DAo. Based on visual assessment, the distribution and incidence of all abnormal hemodynamics did not show any substantial change after Resveratrol treatment.

**TABLE 4:** Incidence of abnormal 4D flow MRI parameters at baseline and follow‐up.

	Native MFS (*n* = 26)			RR MFS (*n* = 20)		
	V1	V2	*p*	V1	V2	*p*
Elevated_velocity						
Inner AAo, *n* (%)	*12 (46%)*	*10 (38%)*	*0.754*	*18 (90%)*	*18 (90%)*	*1.000*
Outer AAo, *n* (%)	*8 (31%)*	*11 (42%)*	*0.453*	*20 (100%)*	*19 (95%)*	*1.000*
Inner arch, *n* (%)	*6 (23%)*	*7 (27%)*	*1.000*	*15 (75%)*	*11 (55%)*	*0.219*
Outer arch, *n* (%)	*5 (19%)*	*9 (35%)*	*0.289*	*16 (80%)*	*11 (55%)*	*0.180*
Inner DAo, *n* (%)	*11 (42%)*	*8 (31%)*	*0.375*	*11 (55%)*	*9 (45%)*	*0.500*
Outer DAo, *n* (%)	*11 (42%)*	*9 (35%)*	*0.687*	*11 (55%)*	*11 (55%)*	*1.000*
Elevated_WSS						
Inner AAo, *n* (%)	*8 (31%)*	*8 (31%)*	*1.000*	*18 (90%)*	*16 (80%)*	*0.500*
Outer AAo, *n* (%)	*4 (15%)*	*5 (19%)*	*1.000*	*19 (95%)*	*18 (90%)*	*1.000*
Inner arch, *n* (%)	*4 (15%)*	*2 (8%)*	*0.625*	*10 (50%)*	*5 (25%)*	*0.180*
Outer arch, *n* (%)	*6 (23%)*	*7 (27%)*	*1.000*	*13 (65%)*	*14 (70%)*	*1.000*
Inner DAo, *n* (%)	*14 (54%)*	*16 (62%)*	*0.754*	*10 (50%)*	*13 (65%)*	*0.250*
Outer DAo, *n* (%)	*8 (31%)*	*10 (38%)*	*0.727*	*7 (35%)*	*10 (50%)*	*0.375*
Abnormally directed velocity						
Inner AAo, *n* (%)	*7 (27%)*	*10 (38%)*	*0.453*	*12 (60%)*	*12 (60%)*	*1.000*
Outer AAo, *n* (%)	*8 (31%)*	*12 (46%)*	*0.219*	*6 (30%)*	*8 (40%)*	*0.727*
Inner arch, *n* (%)	*1 (4%)*	*0 (0%)*	*1.000*	*1 (5%)*	*6 (30%)*	*0.062*
Outer arch, *n* (%)	*0 (0%)*	*2 (8%)*	*0.500*	*0 (0%)*	*5 (25%)*	*0.062*
Inner DAo, *n* (%)	*10 (38%)*	*4 (15%)*	*0.070*	*6 (30%)*	*8 (40%)*	*0.625*
Outer DAo, *n* (%)	*0 (0%)*	*3 (12%)*	*0.250*	*1 (5%)*	*3 (15%)*	*0.500*
Abnormally directed WSS						
Inner AAo, *n* (%)	*15 (58%)*	*16 (62%)*	*1.000*	*20 (100%)*	*20 (100%)*	*NA*
Outer AAo, *n* (%)	*20 (77%)*	*21 (81%)*	*1.000*	*17 (85%)*	*17 (85%)*	*1.000*
Inner arch, *n* (%)	*9 (35%)*	*8 (31%)*	*1.000*	*10 (50%)*	*11 (55%)*	*1.000*
Outer arch, *n* (%)	*6 (23%)*	*8 (31%)*	*0.687*	*12 (60%)*	*15 (75%)*	*0.453*
Inner DAo, *n* (%)	*17 (65%)*	*14 (54%)*	*0.453*	*13 (65%)*	*13 (65%)*	*1.000*
Outer DAo, *n* (%)	*1 (4%)*	*1 (4%)*	*NA*	*2 (10%)*	*4 (20%)*	*0.500*

*Note*: NA, *p*‐values indicate comparisons without discordant pairs. *p*‐values < 0.05 were considered significant (italic and bold italic).

Abbreviations: AAo, ascending aorta; Dao, descending aorta; NA, not applicable; Native MFS, Marfan syndrome without a history of aortic root surgery; RR MFS, Marfan syndrome with a history of aortic root surgery; WSS, wall shear stress.

Raw data was lost in one RR MFS patient, therefore, PWV could not be calculated. Mean PWV (m/s) did not change significantly after 1 year of Resveratrol use for both MFS categories (native MFS: *p* = 0.582, RR MFS: *p* = 0.184). PWV (m/s) was significantly lower for native MFS patients compared to RR MFS at baseline (7.7 ± 1.9 vs. 9.2 ± 1.6), but not at follow‐up (7.9 ± 1.8 vs. 8.2 ± 3.2, *p = 0.707*) (Table S4).

Age was negatively associated with WSS magnitude in the inner (r = −0.45, *n* = 20) and outer arch (r = −0.49, *n* = 20), and inner (r = −0.53, *n* = 20) and outer DAo WSS (r = −0.69, *n* = 20) and with outer DAo velocity in the RR MFS patients (r = −0.61, *n* = 20). Furthermore, a positive correlation of PWV and age was found for RR MFS patients (*r* = 0.59, *n* = 19), but not for native MFS patients (*p* = 0.117). Lastly, change in outer AAo velocity was significantly associated with aortic root growth in native MFS patients (*r* = 0.41, *n* = 26).

No significant correlations between any other of the (change of) 4D flow MRI parameters and aortic growth in the corresponding region were observed (Tables S5–S7).

## 4 Discussion

The main finding of this study is that there were no significant changes in mean velocity, WSS, or PWV in native or RR Marfan patients after 1 year of treatment with Resveratrol.

Geiger and colleagues performed an observational longitudinal 4D flow MRI study in MFS patients and reported generally stable WSS in the thoracic aorta in 3 years of follow‐up [15]. Therefore, changes due to disease progression were not expected in the current study. However, changes in the 4D flow MRI parameters were anticipated, as Resveratrol administration has been shown to modify the elastic properties of the aortic wall in nonhuman primates [31]. The current study results could mean that there is no effect of Resveratrol on aortic wall properties in humans, but it might also mean that the analysis had insufficient sensitivity to detect these changes or that they develop after a longer follow‐up duration.

The presence of an aortic graft is known to influence the local aortic hemodynamics [16]. Indeed, in the current study, higher velocity and WSS values were observed in the outer AAo of RR MFS patients than in native MF patients. This might be explained by the fact that the stiff graft is less compliant compared to the native aortic tissue, thereby losing the Windkessel effect, which results in higher velocities [32]. This hypothesis is supported by a previous study, which found higher peak velocities in the entire aorta of patients with a history of valve sparing aortic root surgery compared to healthy controls [33]. Moreover, a longitudinal study investigating changes before and after valve sparing aortic root surgery in 16 patients with heritable thoracic aortic disease had similar findings [16]. The latter study showed a partial normalization of abnormal flow parameters in the DAo (in‐plane rotational flow and circumferential WSS) after surgery, suggesting that these parameters may play a limited role in the increased risk of type B dissections in RR MFS patients, while other parameters that were abnormal before surgery remained unchanged after surgery (decreased axial WSS and systolic flow reversal). However, as decreased axial WSS and systolic flow reversal were present in both native and RR MFS patients in their study, their role in the elevated risk of type B dissection after surgery is uncertain.

In the current study, by measuring the abnormal flow direction, both abnormal in‐plane rotational flow and abnormal systolic flow reversal were incorporated, which might provide a better overview of the overall abnormalities in blood flow. A decrease in proximal inner DAo WSS of MFS patients has been shown in a previous study [15]. Furthermore, abnormal 4D flow MRI parameter direction and magnitude, as well as vortical flow formation in the proximal inner DAo of MFS patients, have been previously described and have been related to local aortic dilatation [13, 14, 34, 35, 36]. In one of these studies, proximal DAo relative pressure gradients were significantly decreased in MFS patients without local aortic dilatation compared to healthy volunteers [36]. Furthermore, a loss of helical flow in this region has been associated with patients without connective tissue disease experiencing a type B dissection [37]. Indeed, this region of abnormal hemodynamics is a common location of type B dissections [34, 38, 39]. This might be explained by the fact that the ligamentum arteriosum connects to the DAo at this location, restricting local motion, and by the fact that the WSS modifies the release of vasoactive mediators by endothelial cells [14, 34, 36, 38, 40].

In a previous study, it has been proposed that the abnormal blood flow in the DAo in MFS is caused by inefficient blood flow due to sudden geometric or mechanical heterogeneity [34]. In that study, it was concluded that these abnormal flow patterns are most likely caused by differences in arterial stiffness between MFS patients and healthy volunteers. However, the current study data suggest that differences in arterial stiffness alone cannot fully explain this pattern, as a similar pattern of abnormal flow and WSS in the proximal DAo was found for both native and RR MFS patients, but a higher arterial stiffness was found for the RR MFS patients at the time of the baseline MRI examination. Another possible explanation for abnormal flow in the proximal DAo might be the fact that MFS aortas are more tortuous compared to healthy volunteers, resulting in a geometry with a sharp edge in the proximal DAo, which results in inefficient blood flow [41, 42]. It is unlikely that Resveratrol, or any medication treatment, for that matter, changes aortic morphology, which might explain the unchanged incidence maps in the current study. If indeed the aortic morphology causes these abnormal flow patterns and these flow patterns are related to the occurrence of aortic events, this raises the question whether any treatment that does not change the morphology of the aorta would be able to decrease aortic events in the DAo.

PWV, a measure of arterial stiffness, is known to be elevated in MFS patients and increases with disease progression [43]. RR MFS patients have a higher risk for aortic complications [4, 44]. Although RR MFS PWV did not decrease significantly after 1 year of Resveratrol treatment, the PWV was no longer significantly higher than in native MFS patients, which might indicate a beneficial effect of Resveratrol. In a clinical study involving 50 Type‐II diabetic patients, patients received Resveratrol 100 mg/day for three months and showed a decrease in cardio‐ankle vascular index, a blood pressure‐independent measure for arterial stiffness [45]. Moreover, in a study in nonhuman primates with atherosclerosis, a reduced aortic PWV was observed after Resveratrol treatment [31].

Although it has been shown that higher PWV values are associated with an increase in age, an association also observed in the RR MFS patients in the current study, a mean annual increase in PWV in patients with MFS is not yet established [46]. Normal values for PWV per age category have been established in the healthy population [47]. The PWV values for native and RR Marfan patients having a median age of approximately 35 years in the current study correspond to the healthy population values for subjects in their fifties and sixties, respectively. Furthermore, a PWV > 10 m/s is reported to be suggestive of significant alterations of aortic function and increased mortality and cardiovascular risk in patients with arterial hypertension [48]. The higher PWV in the RR MFS patients puts them close to the population at risk, which might partly explain the increased risk of type B dissections within this patient group [4].

### 4.1 Limitations

This study had a short follow‐up duration of 1 year, which might be insufficient to observe long‐term effects of Resveratrol on aortic hemodynamics. Also, the study population was relatively small, limiting the generalizability of the results. Another limitation was the use of a different acceleration scheme for acquiring the 4D flow MRI scans used to create the atlas of age‐ and sex‐matched healthy volunteers. However, we have previously investigated the differences between pseudo‐spiral with compressed sensing and k‐t principal component analysis acquisitions and found comparable velocity and flow measurements between the two methods [19]. Therefore, we do not believe that the use of two different acquisition methods has impacted our results. Furthermore, we did not evaluate cardiac function. However, cardiac function is generally not or at most slightly decreased but still within the normal range in Marfan syndrome compared to healthy volunteers [49]. Therefore, we do not expect that differences in cardiac function had any effect on the observed 4D flow MRI parameters in the current study.

### 4.2 Conclusion

In this sub‐study of a multicenter single‐arm study investigating the effects of Resveratrol on aortic growth and hemodynamics, no significant changes in any 4D flow MRI parameters were found after 1 year.

## Supporting information


**Data S1.** Supporting Information.
