# Supplementary material for: Aortic Function in a Longitudinal 4D Flow MRI Study in Marfan Syndrome Patients Receiving Resveratrol
Source: J Magn Reson Imaging. 2025 Jun 30;62(5):1412–22. doi: 10.1002/jmri.70021 (PMC12507144; doi:10.1002/jmri.70021)
Supplement: Supplementary file 1 — Data S1. Supporting Information. [file JMRI-62-1412-s001.docx]

**Supplementary material.**

**Table 1.** Individual aortic diameter changes per group

|  | **Native MFS**  **(n=26)** | | | | **RR MFS  (n=20)** | | | |
| --- | --- | --- | --- | --- | --- | --- | --- | --- |
| *Aortic diameter*  *(mm)* | Baseline  Mean ± SD | Follow-up  Mean ± SD | Change  Mean [95% CI] | p-value | Baseline  Mean ± SD | Follow-up  Mean ± SD | Change  Mean [95% CI] | p-value |
| Root | 41.4 ± 4.6 | 41.1 ± 4.7 | -0.3 [-0.8, 0.3] | *0.302* | 37.5 ± 3.2 | 37.6 ± 4.0 | 0.1 [-0.6, 0.9] | *0.697* |
| Ascending | 29.2 ± 2.6 | 29.2 ± 2.5 | 0.0 [-0.6, 0.5] | *0.894* | 31.8 ± 2.7 | 31.8 ± 2.4 | 0.0 [-0.7, 0.7] | *1.000* |
| Arch | 24.9 ± 2.1 | 25.2 ± 2.4 | 0.3 [-0.2, 0.7] | *0.238* | 29.0 ± 2.7 | 28.8 ± 2.3 | -0.2 [-0.9, 0.4] | *0.451* |
| Proximal descending | 24.6 ± 2.6 | 24.5 ± 2.6 | -0.1 [-0.6, 0.4] | *0.640* | 27.7 ± 4.1 | 28.0 ± 4.3 | 0.3 [-0.7, 1.4] | *0.482* |
| Distal descending | 20.6 ± 2.1 | 20.7 ± 2.0 | 0.2 [-0.4, 0.7] | *0.538* | 23.4 ± 1.6 | 23.6 ± 1.7 | 0.2 [-0.3, 0.7] | *0.447* |

Native MFS: Marfan syndrome without a history of aortic root surgery, RR MFS: Marfan syndrome with a history of aortic root surgery

|  | **Native MFS**  **(n=26)** | **RR MFS  (n=20)** |  |  |
| --- | --- | --- | --- | --- |
| ***Velocity (m/s)*** | **Mean ± SD** | **Mean ± SD** | **Difference**  **Mean [95% CI]** | **P-value** |
| *Baseline* |  |  |  |  |
| Inner AAo | 0.61 ± 0.11 | 0.68 ± 0.14 | 0.07 [-0.01, 0.15] | *0.075* |
| Outer AAo | 0.50 ± 0.09 | 0.80 ± 0.15 | 0.30 [0.22, 0.38] | ***<0.001*** |
| Inner arch | 0.61 ± 0.09 | 0.62 ± 0.12 | 0.01 [-0.06, 0.08] | *0.764* |
| Outer arch | 0.53 ± 0.09 | 0.65 ± 0.14 | 0.13 [0.06, 0.20] | ***0.001*** |
| Inner DAo | 0.64 ± 0.10 | 0.68 ± 0.15 | 0.04 [-0.03, 0.12] | *0.256* |
| Outer DAo | 0.68 ± 0.12 | 0.68 ± 0.13 | 0.00 [-0.08, 0.07] | *0.932* |
| *Follow-up* |  |  |  |  |
| Inner AAo | 0.61 ± 0.11 | 0.67 ± 0.16 | 0.06 [-0.02, 0.14] | *0.150* |
| Outer AAo | 0.49 ± 0.10 | 0.79 ± 0.14 | 0.29 [0.22, 0.37] | ***<0.001*** |
| Inner arch | 0.61 ± 0.11 | 0.62 ± 0.10 | 0.01 [-0.06, 0.07] | *0.834* |
| Outer arch | 0.53 ± 0.09 | 0.65 ± 0.14 | 0.12 [0.04, 0.19] | ***0.002*** |
| Inner DAo | 0.65 ± 0.10 | 0.67 ± 0.12 | 0.02 [-0.04, 0.09] | *0.463* |
| Outer DAo | 0.69 ± 0.12 | 0.70 ± 0.14 | 0.02 [-0.06, 0.10] | *0.698* |
| *Change* |  |  |  |  |
| Inner AAo | 0.00 ± 0.08 | -0.01 ± 0.12 | -0.01 [-0.07, 0.05] | *0.780* |
| Outer AAo | 0.00 ± 0.06 | -0.02 ± 0.07 | -0.01 [-0.05, 0.03] | *0.543* |
| Inner arch | 0.00 ± 0.08 | 0.00 ± 0.08 | 0.00 [-0.05, 0.04] | *0.885* |
| Outer arch | 0.00 ± 0.08 | -0.01 ± 0.09 | -0.01 [-0.06, 0.04] | *0.662* |
| Inner DAo | 0.01 ± 0.07 | -0.01 ± 0.08 | -0.02 [-0.07, 0.02] | *0.344* |
| Outer DAo | 0.00 ± 0.09 | 0.02 ± 0.07 | 0.02 [-0.03, 0.07] | *0.428* |

**Table 2.** Mean velocity comparison between groups
AAo: Ascending aorta, DAo: Descending aorta, Native MFS: Marfan syndrome without a history of aortic root surgery, RR MFS: Marfan syndrome with a history of aortic root surgery

**Table 3.** Wall shear stress comparison between groups

|  | **Native MFS**  **(n=26)** | **RR MFS  (n=20)** |  |  |
| --- | --- | --- | --- | --- |
| **WSS (m/s)** | **Mean ± SD** | **Mean ± SD** | **Difference**  **Mean [95% CI]** | **P-value** |
| *Baseline* |  |  |  |  |
| Inner AAo | 0.70 ± 0.14 | 0.74 ± 0.21 | 0.03 [-0.08, 0.14] | *0.545* |
| Outer AAo | 0.59 ± 0.15 | 0.95 ± 0.23 | 0.36 [0.24, 0.48] | ***<0.001*** |
| Inner arch | 0.82 ± 0.14 | 0.80 ± 0.20 | -0.02 [-0.13, 0.09] | *0.696* |
| Outer arch | 0.71 ± 0.13 | 0.80 ± 0.21 | 0.10 [-0.01, 0.21] | *0.078* |
| Inner DAo | 1.08 ± 0.21 | 1.02 ± 0.29 | -0.06 [-0.21, 0.10] | *0.454* |
| Outer DAo | 1.09 ± 0.24 | 0.97 ± 0.24 | -0.12 [-0.27, 0.02] | *0.098* |
| *Follow-up* |  |  |  |  |
| Inner AAo | 0.69 ± 0.15 | 0.72 ± 0.23 | 0.03 [-0.09, 0.15] | *0.616* |
| Outer AAo | 0.57 ± 0.15 | 0.91 ± 0.21 | 0.34 [0.23, 0.45] | ***<0.001*** |
| Inner arch | 0.81 ± 0.15 | 0.78 ± 0.15 | -0.04 [-0.13, 0.05] | *0.429* |
| Outer arch | 0.71 ± 0.15 | 0.79 ± 0.17 | 0.08 [-0.01, 0.18] | *0.090* |
| Inner DAo | 1.08 ± 0.17 | 0.98 ± 0.21 | -0.10 [-0.22, 0.02] | *0.089* |
| Outer DAo | 1.08 ± 0.21 | 1.00 ± 0.26 | -0.08 [-0.23, 0.06] | *0.253* |
| *Change* |  |  |  |  |
| Inner AAo | -0.01 ± 0.12 | -0.01 ± 0.20 | 0.00 [-0.10, 0.11] | *0.954* |
| Outer AAo | -0.01 ± 0.08 | -0.04 ± 0.11 | 0.02 [-0.04, 0.08] | *0.442* |
| Inner arch | -0.01 ± 0.12 | -0.02 ± 0.13 | 0.02 [-0.06, 0.09] | *0.680* |
| Outer arch | 0.00 ± 0.13 | -0.01 ± 0.11 | 0.01 [-0.06, 0.09] | *0.738* |
| Inner DAo | 0.01 ± 0.14 | -0.04 ± 0.15 | 0.04 [-0.04, 0.13] | *0.314* |
| Outer DAo | 0.00 ± 0.16 | 0.03 ± 0.13 | -0.04 [-0.12, 0.05] | *0.379* |

*AAo: Ascending aorta, DAo: Descending aorta, Native MFS: Marfan syndrome without a history of aortic root surgery, RR MFS: Marfan syndrome with a history of aortic root surgery, WSS: Wall shear stress*

**Table 4.** Pulse wave velocity comparison between groups

|  | **Native MFS**  **(n=26)** | **RR MFS  (n=19)** |  |  |
| --- | --- | --- | --- | --- |
| **Pulse wave velocity (m/s)** | **Mean ± SD** | **Mean ± SD** | **Difference**  **Mean [95% CI]** | **P-value** |
| Baseline | 7.7 ± 1.9 | 9.2 ± 1.6 | 1.5 [0.5, 2.6] | ***0.006*** |
| Follow-up | 7.9 ± 1.8 | 8.2 ± 3.2 | 0.3 [-1.3, 1.9] | *0.707* |
| Change | 0.2 ± 1.6 | -1.0 ± 3.3 | -1.2 [-0.5, 2.9] | *0.149* |

*Native MFS: Marfan syndrome without a history of aortic root surgery, RR MFS: Marfan syndrome with a history of aortic root surgery*

|  | **Mean velocity (m/s)** | | | |  | **Change velocity (m/s)** | | | |
| --- | --- | --- | --- | --- | --- | --- | --- | --- | --- |
|  | **Native MFS**  **(n=26)** | | **RR MFS**  **(n=20)** | |  | **Native MFS**  **(n=26)** | | **RR MFS**  **(n=20)** | |
|  | **R** | **P-value** | **R** | **P-value** |  | **R** | **P-value** | **R** | **P-value** |
| *Inner AAo* |  |  |  |  |  |  |  |  |  |
| Age (years) | 0.15 | *0.462* | 0.07 | *0.777* |  | 0.11 | *0.604* | 0.15 | *0.536* |
| Growth root (mm/year) | 0.12 | *0.557* |  |  |  | -0.11 | *0.606* | *-* | *-* |
| Growth AAo (mm/year) | -0.08 | *0.706* | -0.23 | *0.340* |  | 0.06 | *0.774* | 0.07 | *0.777* |
| *Outer AAo* |  |  |  |  |  |  |  |  |  |
| Age (years) | -0.03 | *0.901* | 0.171 | *0.171* |  | 0.01 | *0.968* | 0.36 | *0.120* |
| Growth root (mm/year) | 0.01 | *0.957* | *-* | *-* |  | 0.41 | ***0.037*** | *-* | *-* |
| Growth AAo (mm/year) | 0.08 | *0.710* | 0.708 | *0.708* |  | 0.22 | *0.280* | -0.10 | *0.665* |
| *Inner Arch* |  |  |  |  |  |  |  |  |  |
| Age (years) | -0.33 | *0.104* | -0.21 | *0.365* |  | 0.06 | *0.777* | 0.08 | *0.723* |
| Growth arch (mm/year) | 0.02 | *0.910* | 0.18 | *0.462* |  | 0.09 | *0.666* | -0.36 | *0.130* |
| Outer arch |  |  |  |  |  |  |  |  |  |
| Age (years) | -0.22 | *0.286* | -0.43 | *0.059* |  | -0.12 | *0.548* | 0.09 | *0.697* |
| Growth arch (mm/year) | 0.02 | *0.923* | 0.15 | *0.529* |  | 0.16 | *0.435* | -0.45 | *0.055* |
| *Inner DAo* |  |  |  |  |  |  |  |  |  |
| Age (years) | -0.17 | *0.409* | -0.44 | *0.054* |  | -0.33 | *0.100* | 0.00 | *0.992* |
| Growth prox DAo (mm/year) | -0.16 | *0.437* | -0.16 | *0.501* |  | -0.35 | *0.078* | 0.31 | *0.189* |
| Growth dist DAo (mm/year) | -0.10 | *0.644* | -0.23 | *0.339* |  | 0.01 | *0.957* | 0.04 | *0.863* |
| *Outer DAo* |  |  |  |  |  |  |  |  |  |
| Age (years) | -0.32 | *0.109* | -0.61 | ***0.004*** |  | -0.08 | *0.715* | -0.12 | *0.620* |
| Growth prox DAo (mm/year) | -0.15 | *0.477* | -0.01 | *0.972* |  | -0.24 | *0.236* | 0.27 | *0.256* |
| Growth dist DAo (mm/year) | -0.09 | *0.679* | -0.13 | *0.589* |  | 0.08 | *0.703* | 0.32 | *0.169* |

**Table 5.** Correlation analysis of regional velocity

*AAo: Ascending aorta, DAo: Descending aorta, dist : distal, Native MFS: Marfan syndrome without a history of aortic root surgery, RR MFS: Marfan syndrome with a history of aortic root surgery, prox: proximal*

**Table 6.** Correlation analysis of regional wall shear stress

|  | **Mean WSS (Pa)** | | | |  | **Change WSS (Pa)** | | | |
| --- | --- | --- | --- | --- | --- | --- | --- | --- | --- |
|  | **Native MFS**  **(n=26)** | | **RR MFS**  **(n=20)** | |  | **Native MFS**  **(n=26)** | | **RR MFS**  **(n=20)** | |
|  | **R** | **P-value** | **R** | **P-value** |  | **R** | **P-value** | **R** | **P-value** |
| *Inner AAo* |  |  |  |  |  |  |  |  |  |
| Age (years) | 0.20 | *0.329* | 0.04 | *0.871* |  | 0.09 | *0.649* | 0.05 | *0.828* |
| Growth root (mm/year) | 0.05 | *0.802* | - | *-* |  | -0.06 | *0.786* | - | *-* |
| Growth AAo (mm/year) | -0.02 | *0.911* | -0.10 | *0.663* |  | -0.04 | *0.832* | 0.11 | *0.641* |
| *Outer AAo* |  |  |  |  |  |  |  |  |  |
| Age (years) | -0.04 | *0.842* | -0.32 | *0.169* |  | -0.06 | *0.771* | 0.21 | *0.375* |
| Growth root (mm/year) | -0.04 | *0.828* | *-* | *-* |  | 0.49 | ***0.010*** | - | *-* |
| Growth AAo (mm/year) | 0.17 | *0.415* | 0.11 | *0.655* |  | 0.18 | *0.391* | -0.30 | *0.195* |
| *Inner Arch* |  |  |  |  |  |  |  |  |  |
| Age (years) | -0.26 | *0.193* | -0.45 | ***0.049*** |  | -0.18 | *0.392* | 0.13 | *0.587* |
| Growth arch (mm/year) | -0.13 | *0.513* | 0.13 | *0.600* |  | 0.26 | *0.191* | -0.18 | *0.471* |
| *Outer arch* |  |  |  |  |  |  |  |  |  |
| Age (years) | -0.19 | *0.344* | -0.49 | ***0.029*** |  | -0.21 | *0.308* | 0.12 | *0.620* |
| Growth arch (mm/year) | -0.02 | *0.926* | 0.02 | *0.927* |  | 0.16 | *0.435* | -0.22 | *0.359* |
| *Inner DAo* |  |  |  |  |  |  |  |  |  |
| Age (years) | -0.20 | *0.320* | -0.53 | ***0.016*** |  | -0.29 | *0.150* | 0.06 | *0.815* |
| Growth prox DAo (mm/year) | -0.09 | *0.651* | -0.20 | *0.393* |  | -0.22 | *0.277* | 0.36 | *0.122* |
| Growth dist DAo (mm/year) | -0.12 | *0.559* | -0.21 | *0.377* |  | 0.16 | *0.435* | 0.06 | *0.795* |
| *Outer DAo* |  |  |  |  |  |  |  |  |  |
| Age (years) | -0.36 | *0.072* | -0.69 | ***0.001*** |  | -0.08 | *0.693* | -0.09 | *0.701* |
| Growth prox DAo (mm/year) | -0.15 | *0.477* | -0.10 | *0.687* |  | -0.08 | *0.690* | 0.28 | *0.234* |
| Growth dist DAo (mm/year) | -0.01 | *0.955* | -0.16 | *0.490* |  | 0.16 | *0.429* | 0.42 | *0.066* |

*AAo: Ascending aorta, DAo: Descending aorta, dist : distal, Native MFS: Marfan syndrome without a history of aortic root surgery, RR MFS: Marfan syndrome with a history of aortic root surgery, prox: proximal*

**Table 7.** Correlation analysis of pulse wave velocity

|  | **Mean PWV (m/s)** | | | |  | **Change PWV m/s)** | | | |
| --- | --- | --- | --- | --- | --- | --- | --- | --- | --- |
|  | **Native MFS**  **(n=26)** | | **RR MFS**  **(n=19)** | |  | **Native MFS**  **(n=26)** | | **RR MFS**  **(n=19)** | |
|  | **R** | **P-value** | **R** | **P-value** |  | **R** | **P-value** | **R** | **P-value** |
| Age (years) | 0.31 | *0.117* | 0.59 | ***0.006*** |  | -0.01 | *0.960* | -0.39 | *0.088* |
| Growth root (mm/year) | 0.00 | *0.986* | - | *-* |  | -0.08 | *0.715* | - | *-* |
| Growth AAo (mm/year) | -0.27 | *0.182* | -0.17 | *0.462* |  | -0.05 | *0.810* | 0.02 | *0.928* |
| Growth arch (mm/year) | 0.02 | *0.921* | 0.31 | *0.199* |  | -0.10 | *0.639* | -0.13 | *0.609* |
| Growth prox DAo (mm/year) | -0.10 | *0.618* | 0.01 | *0.950* |  | -0.06 | *0.766* | 0.02 | *0.919* |
| Growth dist DAo (mm/year) | -0.03 | *0.868* | -0.23 | *0.331* |  | -0.30 | *0.140* | 0.12 | *0.600* |

*AAo: Ascending aorta, DAo: Descending aorta, dist : distal, Native MFS: Marfan syndrome without a history of aortic root surgery, RR MFS: Marfan syndrome with a history of aortic root surgery, prox: proximal*
